# Prospective single-arm study of 72 Gy hyperfractionated radiation therapy and combination chemotherapy for anaplastic astrocytomas

**DOI:** 10.1186/1471-2407-8-11

**Published:** 2008-01-16

**Authors:** Takuma Nomiya, Kenji Nemoto, Toshihiro Kumabe, Yoshihiro Takai, Shogo Yamada

**Affiliations:** 1 Department of Radiation Oncology, Tohoku University School of Medicine, Sendai, Japan; 2 Department of Neurosurgery, Tohoku University School of Medicine, Sendai, Japan

## Abstract

**Background:**

Despite intensive multimodal treatment, outcome of patients with malignant glioma remains poor, and a standard dose of radiotherapy for anaplastic astrocytoma has not been defined. In the past RTOG study (83-02), the arm of 72 Gy hyperfractionated radiotherapy (HFRT) for malignant gliomas showed better outcome than the arms of higher doses (76.8 – 81.6 Gy) and the arms of lower doses (48 – 54.4 Gy). The purpose of this study is to verify the efficacy of this protocol.

**Methods:**

From July 1995, 44 consecutive eligible patients with histologically proven anaplastic astrocytoma were enrolled in this study (HFRT group). The standard regimen in this protocol was post-operative radiotherapy of 72 Gy in 60 fractions (1.2 Gy/fraction, 2 fractions/day) with concurrent chemotherapy (weekly ACNU). The primary endpoint was local control rate (LCR), and the secondary endpoints were overall survival (OS), progression-free survival (PFS) and late toxicity.

**Results:**

Three-year OS of the HFRT group was 64.8% (95% confidence interval; 48.4–81.3%). Three-year PFS rate and LCR were 64.4% (95%CI: 48.4–80.3%) and 81.6% (95%CI: 69.2–94.8%), respectively.

The number of failures at 5 years in the HFRT group were 14 (32%). The number of failures inside the irradiation field was only about half (50%) of all failures. One (2%) of the patients clinically diagnosed as brain necrosis due to radiation therapy.

**Conclusion:**

The results of this study suggested that 72 Gy HFRT seemed to show favorable outcome for patients with anaplastic astrocytoma with tolerable toxicity.

## Background

Despite the availability of combined multimodality treatment, anaplastic astrocytoma (AA) has an unfavorable prognosis. Although radical surgery and radiation therapy have been performed, loco-regional controllability has not been improved. It has been reported that the median survival period of patients with AA is 20 – 40 months (approximately 30 months on average) [1-5]. The major prognostic factors have been reported to be age, Karnofsky performance status and site of lesion, and the minor prognostic factors have been reported to be extent of surgery, total radiation dose, Ki67 labeling index, and various other factors [1-9].

In radiation therapy, dose escalation by conventional fractionation has been unsuccessful, and studies on dose escalation by hyperfractionated or accelerated hyperfractionated radiation therapy have been performed [1,10]. No significant difference was found between survival in the low-dose group (60 – 72 Gy) and survival in the high-dose group (74 – 82 Gy), but results suggested that 72 Gy was the best dose for good prognosis. Based on results of those studies, we planned a single-arm study of 72 Gy hyperfractionated radiation therapy limited to patients with grade 3 glioma.

The primary objective of this study was to evaluate the efficacy of 72 Gy hyperfractionated radiation therapy in terms of overall survival, progression-free survival and loco-regional control rate for patients with AA. The secondary objective was to evaluate the toxicity and feasibility of this treatment.

## Methods

### Patients

Since July 1995, patients with histologically proven grade 3 glioma (anaplastic astrocytoma) have been treated in our institution by 72 Gy hyperfractionated radiation therapy (HFRT) with surgery and chemotherapy. Histological diagnosis was determined on the basis of the 2nd edition of WHO (World Health Organization) classification of brain tumors.

The main points of eligibility criteria of this study were 1) histopathologically proven anaplastic astrocytoma, 2) primary lesion arising from the central nervous system (except brain stem and spinal cord), 3) feasibility of completion of a course of treatment, and 4) written informed consent for surgery and verbal informed consent for radiotherapy and chemotherapy having been obtained. The treatment protocol has been inspected and validity of treatment has been approved by the Institutional Ethics Committee of Tohoku University School of Medicine.

All patients with anaplastic astrocytoma who met the above criteria were enrolled in this study. Patients with other grade 3 gliomas (anaplastic oligoastrocytoma, anaplastic oligodendroglioma, etc.) were excluded in order to exclude histopathological bias and influence on clinical outcome. Patients with grade 3 glioma arising from the brain stem or spinal cord were excluded from this study because of intolerability for high-dose radiation therapy. Patients with diffusely extended gliomatosis cerebri requiring nearly whole brain irradiation were also excluded because of intolerability for high-dose whole brain irradiation. Patients who appeared to be in extremely poor condition (Karnofsky score ≤ 30, indication of emergency operation, and definitely unable to complete a course of treatment) were excluded from this study. Informed consent for the treatment was obtained from all enrolled patients.

Performance status (PS) was determined according to the criteria of Karnofsky score before treatment. Tumor size was measured as the length of the long axis of the tumor by more than one diagnostic radiologist using images of enhanced-computed tomography (CT) and/or T2-weighted (T2WI) or enhanced T1-weighted (T1WI) magnetic resonance (MR) imaging.

### Radiation therapy

HFRT group: All patients were irradiated with 6–10 mega-volt photons by a linear accelerator, and all patients were immobilized by a resinous shell during irradiation. Patients were irradiated with an extended local field during treatment, and none of the patients underwent whole brain irradiation. The extended local irradiation field included the whole T2WI high-intensity region on MR images or a 2–3-cm margin around the tumor or tumor bed, and more than 2 fields were used. The standard schedule of irradiation was 72 Gy/60 fractions/6 weeks (1.2 Gy/fraction, and 2 fractions in a day with an interval of at least 6 hours).

Biologically equivalent dose (BED) was calculated on the basis of the Linear-Quadratic model (a). According to previously reported experimental data, the alpha/beta value of grade 3 gliomas was assumed to be 14 Gy [11-13].

BED (Dx) = [(alpha/beta + dx)/(alpha/beta + dr)] × Dr ---(a)

(Dr = total reference dose, dr = single reference dose/fraction, Dx = BED of altered fractionation, dx = altered single dose/fraction.)

### Other treatments

The extent of surgery was evaluated by postoperative MR imaging within 72 hours after surgery (All patients have routinely been examined by MR imaging.). If the tumor was enhanced on preoperative MR images, gross total resection of the tumor was defined as resection with no residual enhanced tumor, subtotal resection was as defined as more than 75% resection, and partial resection was as defined as less than 75% resection. If the tumor was not enhanced on preoperative MR images, resection was evaluated on the basis of resection of the high-intensity lesion on T2-weighted MR images. Surgery (or biopsy) was performed before radiation therapy, and chemotherapy was performed concomitantly. The main drug used in chemotherapy was nimustine hydrochloride (ACNU). Patients were given 2–3 weekly courses of 2–3 mg/kg ACNU (single injection per week). The dose of chemotherapy was determined according to patient's age, renal function and general condition.

### Toxicity

Toxicity was clinically diagnosed on the basis of CTC (common toxicity criteria) version 2.0. Patients who showed severe adverse events (≥ grade 3 leukoencephalopathy-associated radiological findings, brain necrosis diagnosed by CT, MR imaging and/or histopathologic findings) were regarded as having late toxicity. After a course of treatment, follow-up CT or MRI was performed once in 1 to 4 months. If a new enhanced region appeared in the follow-up examination but improved with no treatment during the observation period, it was regarded as post-operative change or no recurrence. If a new enhanced region or tumorous lesion appeared and there were definite findings of progression, it was regarded as a failure case. If it was difficult to distinguish failure from brain necrosis, biopsy was performed and histopathological proof was obtained.

### Statistical analysis

Survival period was calculated from the first date of treatment, and the final follow-up date was October 1, 2004. Survival curves were analyzed by the Kaplan-Meier method and the logrank test. Overall survival was calculated as the period to the date of death, progression-free survival was calculated as the period to the date of the first progression (loco-regional failure and/or distant failure), and local control survival was calculated as the period to the date of the first loco-regional progression (distant metastasis and dissemination outside the irradiation field not included).

## Results

From July 1995 to August 2004, 56 patients were initially diagnosed as having anaplastic astrocytoma. It took 9 years to enroll a sufficient number of patients, but the number of patients per year approximately corresponds to the calculated incidence [incidence of AA in our area = 6/year; 12/100,000 (a) × 0.28 (b) × 0.18 (c) × 1,000,000 (d) = 6 (a: incidence of brain tumor in Japan, b: rate of glioma in all brain tumors, c: rate of AA in all gliomas, d: population of the area; the Ministry of Health and Welfare, Japan, 1998)]. Three (5.3%) of 56 patients were excluded from this trial because of intolerability of normal tissue (tumor located at basal ganglia or gliomatosis cerebri). One patient (1.7%) was histologically reviewed and classified into glioblastoma multiforme. Eight (14.3%) of 56 patients were excluded because informed consent could not be obtained or they chose other treatment protocols.

A total of 44 eligible patients with histologically proven anaplastic astrocytoma underwent 72 Gy hyperfractionated radiation therapy. All patients completed the scheduled irradiation. The median follow-up period of survival patients was 35.1 months. Characteristics of the patients are shown in Table 1.

**Table 1:** Patients' characteristics

Number of patients		44
Age	(mean ± S.D.)	39.8 ± 17.3

Gender	Male	30
	Female	14

Karnofsky score (performance status)	80–100	27 (61%)
	60–70	15 (34%)
	40–50	2 (5%)
	0–30	0

Site of lesion	Frontal lobe	23 (52%)
	Temporal lobe	9 (21%)
	Parietal lobe	3 (7%)
	Others	9 (21%)

Tumor size (cm)	median (range)	5 cm (1–11)

Extent of surgery	Total/Subtotal resection	23 (52%)
	Partial resection/Biopsy only	21 (48%)

BED (Gy)	(mean ± S.D.)	68.5 ± 0.36

Chemotherapy	with	36 (82%)
	without	8 (18%)

**Figure 1:**
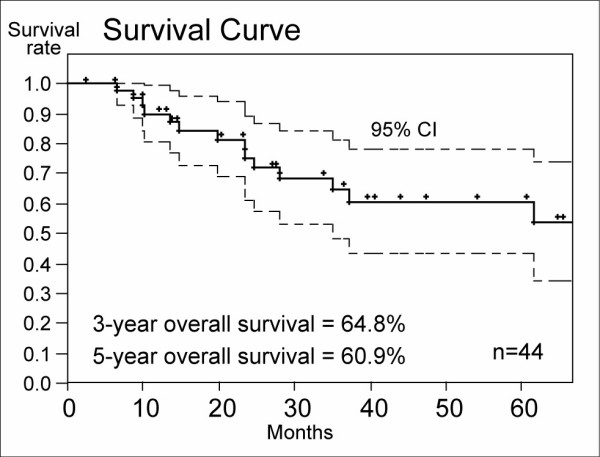
Overall survival rates of the HFRT group. Three-year and 5-year overall survival rates were 64.8% and 60.8%, respectively.

**Figure 2:**
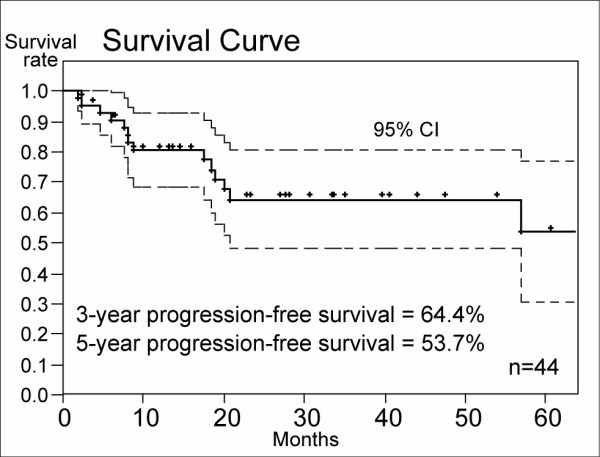
Progression-free survival rates of the HFRT group. Three-year and 5-year PFS rates were 64.4% and 53.7%, respectively.

**Figure 3:**
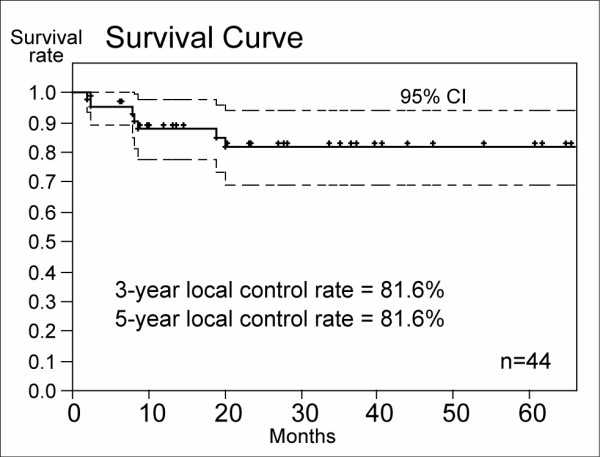
Local control rate of the HFRT group. Three-year and 5-year LCRs were 81.6% and 81.6%, respectively.

Figure 1 shows the overall survival curve of the 44 patients. Three-year overall survival rate of the HFRT group was 64.8% (95% confidence interval: 48.4–81.3%) and 5-year overall survival rate was 60.9% (95%CI: 43.6 – 71.2%). Three-year and 5-year PFS rates were 64.4% (95%CI: 48.4% – 80.3%) and 53.7% (95% CI: 30.4 – 77.1%), respectively (Figure 2). Three-year and 5-year LCRs were 81.6% (69.2 – 94.8%: 95%CI) and 81.6% (95%CI: 69.2–94.8%), respectively (Figure 3).

Patterns of failure are shown in Table 2. The number of failures at 5 years in the HFRT group was 14 (32%). The number of failures inside the irradiation field was only about half (7/14: 50%) of all failures. On the other hand, the ratio of failures outside the irradiation field was higher (10/14: 71%) than those inside field. Nine (90%) of the 10 cases of failure outside the irradiation field were cerebrospinal dissemination that was diagnosed by enhanced-CT/MRI. The other patient showed diffuse infiltration like gliomatosis cerebri.

**Table 2:** Patterns of failure and severe late toxicity

Total failure	14/44 (32%)
Failure inside field	7/14 (50%)
Failure outside field	10/14 (71%) (CSF dissemination: 9, contralateral brain: 1)
Severe late toxicity	
Brain necrosis	1 (2%)

All 44 patients completed the scheduled irradiation (72 Gy/60 fr./6 weeks) and 39 (89%) of the 44 patients completed radiation therapy within a 4-day prolongation (including holidays). Five patients (11%) took more than 4-day prolongation in radiation therapy. In 4 of those 5 patients, radiation therapy was suspended due to transient brain edema caused by irradiation or post-operative changes. However, their condition was improved by anti-edema therapy and radiation therapy was restarted as soon as possible. One patient had progression completely outside the irradiation field (contralateral brain) during the radiation therapy. This lesion was not detected in the pre-treatment examinations, so the lesion was diagnosed by biopsy and the radiation therapy was suspended during the examination. Finally, this patient was diagnosed as having progression in the course of treatment, but, this patient was included in the analysis due to the initial condition and completion of treatment. This patient was censored as a case of failure outside the irradiation field. There were no other cases of suspension of radiation therapy due to acute toxicity > grade 3.

Six (14%) of the 44 patients were not given chemotherapy due to their age (> 60 years). Two patients (5%) were not so old but they were not given chemotherapy because of their poor general condition. Thirty-six patients (82%) were given at least 2 courses of weekly ACNU infusion.

One (2%) of the patients in the HFRT group showed severe late toxicity clinically diagnosed as brain necrosis due to radiation therapy. This patient had undergone intra-thecal injection of MTX (Due to residual tumor and possibility of dissemination, MTX was used only in this case.). Radiographical change appeared 3 months after radiation therapy, and the patient died of cerebral infarction 30 months after the radiographical change had appeared. No definite relationship between death and late toxicity was found, and the case was therefore considered to be non-cancer-related and non-treatment-related death. Two patients showed mild changes in radiographical findings with no symptoms. It was not determined whether the changes were local failure or brain necrosis, and the patients are now being observed with no treatment at the date of final follow-up. Other patients showed no significant changes in clinical symptoms after irradiation.

## Discussion

Werner-Wasik et al. analyzed cases of 130 AA in an RTOG 83-02 trial and they reported that the median survival time (MST) of patients with AA was 40.3 months [1]. In the RTOG 83-02 trial, it was found that the MSTs of the 48–54.4 Gy, 64.8–72 Gy and 76.8–81.6 Gy groups were 35–40.6, 50–85.8 and 30.4–35.4 months, respectively. These results showed that a very small dose and a very large dose of radiation therapy did not lead to a favorable prognosis. The 64.8 Gy group showed a very long MST (85.8 months), but this group consisted of only 9 patients and data are therefore not sufficient.

Tortosa et al. analyzed 95 cases of anaplastic glioma retrospectively, and MST of the patients was 29 months [2]. The median age of the patients was 49 years, the proportion of patients with KPS ≥ 80 was 60%, total resection rate was 35% and standard dose of external beam radiation therapy (EBRT) was 60 Gy/30 fr. According to known prognostic factors, the advance age of patients and lower total resection rate in this trial are thought to be the reasons for the short MST.

Levin et al. analyzed 90 cases of anaplastic glioma (including 69 cases of AA) in their phase II study of accelerated hyperfractionated radiation therapy with chemotherapy, and the results showed that the MST of patients with AA was 27.8 months [3]. The patients' characteristics in that study were not so poor (median age: 37 years, proportion of patients with KPS ≥ 90: 77%, total or subtotal resection rate: 77%). However, the EBRT is schedule was irregular (60 Gy/t.i.d. with 2-week intervals) and the irradiated dose did not reach 60 Gy (55–57 Gy: 54.4%) in more than half of the patients. The insufficient dose of EBRT might be one of the reasons for the relatively poor outcome. Jeremic et al. analyzed 12 cases of AA and Urtasun et al. analyzed 21 cases of AA, and the MSTs of the patients in their studies were 42 months and 38 months, respectively [14,15]. However, both of those studies have a small impact due to the small number of patients.

Prados et al. analyzed 110 cases of anaplastic glioma (including 107 cases of AA) and they reported that the 2-year and 5-year survival were 80% and 66%, respectively [16]. The characteristics of patients in their trial were favorable (median age: 37 years, total and subtotal resection rate: 72%, median KPS: 90, EBRT: 60 Gy/30 fr., with chemotherapy). Although the KPS and the extent of surgery those are known as significant prognostic factors are better than those of the present study, the outcome of the present study is almost similar to that of their study. One of the reasons that the outcome of the present study is almost equal to that by Prados et al. despite the disadvantage in patients' characteristics might be due to benefits from fractionation and total dose of radiation therapy. The dose of EBRT in that trial was less than that in the present study (60 Gy/30 fr. vs. 72 Gy/60 fr.). On the basis of the outcomes of above-mentioned reports, it is suggested that not only extent of surgery but also radiation dose has influence on patients' prognosis. EBRT of 60 Gy seems to be a necessary dose to control AA. The results of above-mentioned reports including RTOG study suggests that the outcome of the patients who underwent radiotherapy with a total dose of less than 60 Gy of EBRT seems to be poorer than that of the patients who underwent radiotherapy with a total dose of ≥ 60 Gy. However, in conventional fractionation (2 Gy/fr.), the risk of brain necrosis much increases when total radiation dose exceed 60 Gy. One of the merits is that HFRT can deliver more total dose with low risk of adverse effect (e.g. brain necrosis) than conventional fractionation.

The major pattern of failure in past studies was loco-regional failure, and it has generally been considered that the controllability of malignant glioma was extremely poor in spite of multimodal treatments [17-19]. In those studies, rates of loco-regional failure were about 80–90% of all failures. In the present study, 5-year local control rate is 81.6% (local failure at 5 years is 18.4%). It is thought that local controllability of AA is getting improved due to advances in resectability and improvement of radiation dose and fractionation.

The average BED of external beam radiation therapy in the HFRT group was 68.5 Gy. It seemed that the higher total dose of radiation therapy contributed to loco-regional controllability. From the viewpoint of radiosensitivity, the alpha/beta value of malignant glioma tends to be larger than that of low grade glioma, and it therefore seems to be possible to deliver high-dose irradiation to the tumor bed with a localized irradiation field by a hyperfractionated regimen and to be possible to reduce toxicity of normal tissue by decreasing single-dose/fraction. The results of the present study suggested that high dosage of irradiation is one of the factors enabling control of grade 3 gliomas. Although there has been a trial using higher single-dose/fraction, higher single-dose/fraction does not seem to be very effective from the viewpoint of alpha/beta values of tumor tissue and normal central nervous tissue [11-13].

The rate of severe late toxicity in this trial was not so high and seemed to be tolerable (Table 2). One of the patients who was diagnosed as having brain necrosis was alive at the last follow-up examination, and the other patient, who died of cerebral infarction, survived for about 30 months after the appearance of toxicity. Up to the time of the last follow-up examination, there was no treatment-related death in this trial. Excessive irradiation and combination of high-dose MTX injection seem to induce brain necrosis [20]. Combination of MTX injection with radiation therapy should therefore be carefully determined.

A new problem is failure outside the irradiation field. The rate of failure outside the field was relatively high in this trial. One possible reason for this is the low rate of loco-regional failure and the large number of long-term survivors. It seems that some of the objectives such as improvement of local controllability and improvement of survival were accomplished by high-dose irradiation to a localized field. If whole brain irradiation is combined, the rate of dissemination outside the local field may decrease, but the risk of toxicity will also increase.

## Conclusion

The results of this study suggested that 72 Gy HFRT seemed to show favorable outcome for patients with anaplastic astrocytoma with tolerable toxicity. Further study is needed to determine whether whole brain irradiation is necessary and what dose is optimal.

## Competing interests

The author(s) declare that they have no competing interests.

## Authors' contributions

TN is corresponding author of this manuscript, participated in clinical work and data analysis as a radiation oncologist, and prepared this manuscript, TK is a main neurosurgeon of this study and participated in clinical work and analysis, NK, YT and SY participated in clinical work, study design and discussion as radiation oncologists. All authors read and approved the final manuscript.

## Pre-publication history

The pre-publication history for this paper can be accessed here:


